# Cost-of-illness studies in nine Central and Eastern European countries

**DOI:** 10.1007/s10198-019-01066-x

**Published:** 2019-05-18

**Authors:** Valentin Brodszky, Zsuzsanna Beretzky, Petra Baji, Fanni Rencz, Márta Péntek, Alexandru Rotar, Konstantin Tachkov, Susanne Mayer, Judit Simon, Maciej Niewada, Rok Hren, László Gulácsi

**Affiliations:** 1 Department of Health Economics, Corvinus University of Budapest, Fővám tér 8., 1093 Budapest, Hungary; 2 Hungarian Academy of Sciences, Premium Postdoctoral Research Program, Nádor u. 7, 1051 Budapest, Hungary; 3 Department of Social Medicine, University of Amsterdam, Meibergdreef 9, 22660, 1100 DD Amsterdam, The Netherlands; 4 Department of Social Pharmacy and Pharmacoeconomics, Faculty of Pharmacy, Medical University of Sofia, 2, Dunav str., 1000 Sofia, Bulgaria; 5 Department of Health Economics, Center for Public Health, Medical University of Vienna, Kinderspitalgasse 15/1, Vienna, 1090 Austria; 6 Department of Experimental and Clinical Pharmacology, Medical University of Warsaw, Banacha 1b, 02-097 Warsaw, Poland; 7 Institute of Mathematics, Physics, and Mechanics, Jadranska 19, 1000 Ljubljana, Slovenia; 8 Department of Physics, University of Ljubljana, Jadranska 19, 1000 Ljubljana, Slovenia; 9 Doctoral School of Business and Management, Corvinus University of Budapest, Fővám tér 8., 1093 Budapest, Hungary; 10 Ludwig Boltzmann Institute Applied Diagnostics, Währinger Gürtel 18-20, Vienna, 1090 Austria

**Keywords:** Cost-of-illness, Disease burden, Central and Eastern Europe, Austria, Bulgaria, The Czech Republic, Croatia, Hungary, Poland, Romania, Slovakia, Slovenia, I10

## Abstract

**Background:**

To date, a multi-country review evaluating the cost-of-illness (COI) studies from the Central and Eastern European (CEE) region has not yet been published. Our main objective was to provide a general description about published COI studies from CEE.

**Methods:**

A systematic search was performed between 1 January 2006 and 1 June 2017 in Medline, EMBASE, The Cochrane Library, CINAHL, and Web of Science to identify all relevant COI studies from nine CEE countries. COI studies reporting costs without any restrictions by age, co-morbidities, or treatment were included. Methodology, publication standards, and cost results were analysed.

**Results:**

We identified 58 studies providing 83 country-specific COI results: Austria (*n* = 9), Bulgaria (*n* = 16), Croatia (*n* = 3), the Czech Republic (*n* = 10), Hungary (*n* = 24), Poland (*n* = 11), Romania (*n* = 3), Slovakia (*n* = 3), and Slovenia (*n* = 4). Endocrine, nutritional, and metabolic diseases (18%), neoplasms (12%), infections (11%), and neurological disorders (11%) were the most frequently studied clinical areas, and multiple sclerosis was the most commonly studied disease. Overall, 57 (98%) of the studies explicitly stated the source of resource use data, 45 (78%) the study perspective, 34 (64%) the costing method, and 24 (58%) reported at least one unit costs. Regardless of methodological differences, a positive relationship was observed between costs of diseases and countries’ per capita GDP.

**Conclusions:**

Cost-of-illness studies varied considerably in terms of methodology, publication practice, and clinical areas. Due to these heterogeneities, transferability of the COI results is limited across Central and Eastern European countries.

**Electronic supplementary material:**

The online version of this article (10.1007/s10198-019-01066-x) contains supplementary material, which is available to authorized users.

## Introduction

Cost-of-illness (COI) studies provide information on the economic burden of a specific disease from a societal, public payer, family or individual perspective. They aim to evaluate not only the disease-related healthcare costs but also the overall costs to society, including both medical and non-medical costs. COI studies can aid the understanding of the importance of a health problem, estimate the main cost components and the cost structure, and, thus, provide valuable cost estimates for use in full economic evaluations [1]. As a result, COI studies are an important type of health economic analysis aiming to support health policy and financing decision-making processes [2]. Over the past decade, health technology assessment has been implemented in most Central and Eastern European (CEE) countries, which, in turn, necessitates reliable, local country-specific COI studies [3–5].

There are no gold standard methods for calculating COI estimates [6–8]. Although standardization of the methods used in COI studies is becoming more and more important to allow comparability, studies apply different designs, methodologies, perspectives, and costing approaches [9, 10]. Until now, several systematic reviews of COI studies have been conducted; however, most of them were focusing on one specific disease. Few reviews targeted a single specific cost item or component, such as informal care, direct medical costs, productivity loss, a specific geographic area, or a specific methodological aspect [10–13]. Nonetheless, COI studies from CEE countries have not been reviewed to date, with the exception of Austria [13].

This review has been undertaken to provide a description of the COI studies in nine CEE countries, namely Austria, Bulgaria, the Czech Republic, Croatia, Hungary, Poland, Romania, Slovakia, and Slovenia, in the past 10 years. The main objectives were to describe study characteristics, methodology, and the COI estimates reported. First, we provide an overview of applied methods. Then, we present and compare the COI estimates across CE countries.

## Methods

### Search strategy

We conducted a systematic review following the PRISMA statement [14]. A literature search was performed using Medline, EMBASE, The Cochrane Library, CINAHL, and Web of Science databases to identify studies that report data on the cost of a disease. The search strategy was based on the keyword “cost of illness” and the name of the given CEE country (online Appendix 1). The search was limited to studies published in the past 10 years (1 January 2006—31 October 2016) and was updated on 30 June 2017 to shorten the time between the end of the search period and publication date. No language restrictions were applied. A complementary, non-systematic literature search was conducted in three countries. Three authors (SM, KT, and ZB) hand-searched for further papers in selected, peer-reviewed, non-indexed local journals in Austria, Bulgaria, and Hungary. The review protocol was not registered.

### Study selection

After removing duplicates, titles and abstracts of studies were reviewed independently by ZB, VB, and LG, and were retrieved if at least one of the reviewers considered the study to be relevant. First, abstracts (publication type) and reviews (publication type) were excluded. Full-text papers of the remaining studies were reviewed and included (ZB, VB, and LG). Any disagreement between reviewers was solved by discussions among the authors to reach consensus.

Studies were selected for further analysis if they met the following inclusion criteria: (i) COI data included for a specific disease without major restriction on the patient population, e.g., by age, co-morbidity, complication, or treatment, (ii) full-text paper, (iii) original research, and (iv) the study population was recruited in Austria, Bulgaria, the Czech Republic, Croatia, Hungary, Poland, Romania, Slovakia, or Slovenia. Studies were not selected for further analysis if they represented clinical trials, reviews, cost-effectiveness studies, budget impact analyses, treatment-related (drug) studies, costs of health programs (e.g., screening), or studies enrolling a patient population with co-morbidities (e.g., diabetic patient with depression).

### Data extraction

A Microsoft Excel spreadsheet was developed to extract data from the identified studies, including general characteristics of the study (year of publication, geographical location, language, and funding source), methodological details of the study (disease, data collection method, study design, setting, costing year, currency, and perspective and costing methods), and results (direct costs, indirect costs, and total costs in euros). The list of extracted variables was created based on health economic checklists and adjusted by screening of six (10%) random articles [6, 15]. Costs reported in currencies other than euro were converted to euro at a mean annual exchange rate, and all costs were inflated to 2017 prices using the harmonised consumer price index extracted from Eurostat [16]. To facilitate cross-country comparisons, costs were also described as a percentage of 2017 GDP per capita. Diseases were categorised according to the International Statistical Classification of Diseases and Related Health Problems 10th Revision (ICD-10 Version:16) [17]. Data extraction was conducted by ZB and respective authors for national languages and double-checked.

## Results

### Study selection

As can be seen from Fig. S1 (online Appendix), after removing 246 duplicates, the search in the electronic databases resulted in 607 potentially relevant papers. Of these studies, 55 were not full-text papers and 98 were reviews. Furthermore, 282 papers did not report disease-related costs, 54 focused on costs of multiple diseases, and 67 focused on the cost of a certain treatment. Overall, 50 articles from the electronic search fulfilled the inclusion criteria. The supplementary local search resulted in another eight relevant articles in non-indexed, peer-reviewed journals (Austria: *n* = 2, Bulgaria: *n* = 5, and Hungary: *n* = 1).

Altogether, we included 58 articles (involving also multi-country studies) that reported results for Hungary (*n* = 24), Bulgaria (*n* = 16), Poland (*n* = 11), Czech Republic (*n* = 10), Austria (*n* = 9), Slovenia (*n* = 4), Croatia (*n* = 3), Slovakia (*n* = 3), and Romania (*n* = 3).

Thirteen additional COI studies did not meet to our eligibility criteria (e.g., involved samples restricted by age, co-morbidity, complication, or treatment), but we found their results worthy of attention, and hence, a summary of their characteristics and main results is presented in online Appendix 1.

### Study characteristics

**Fig. 1:**
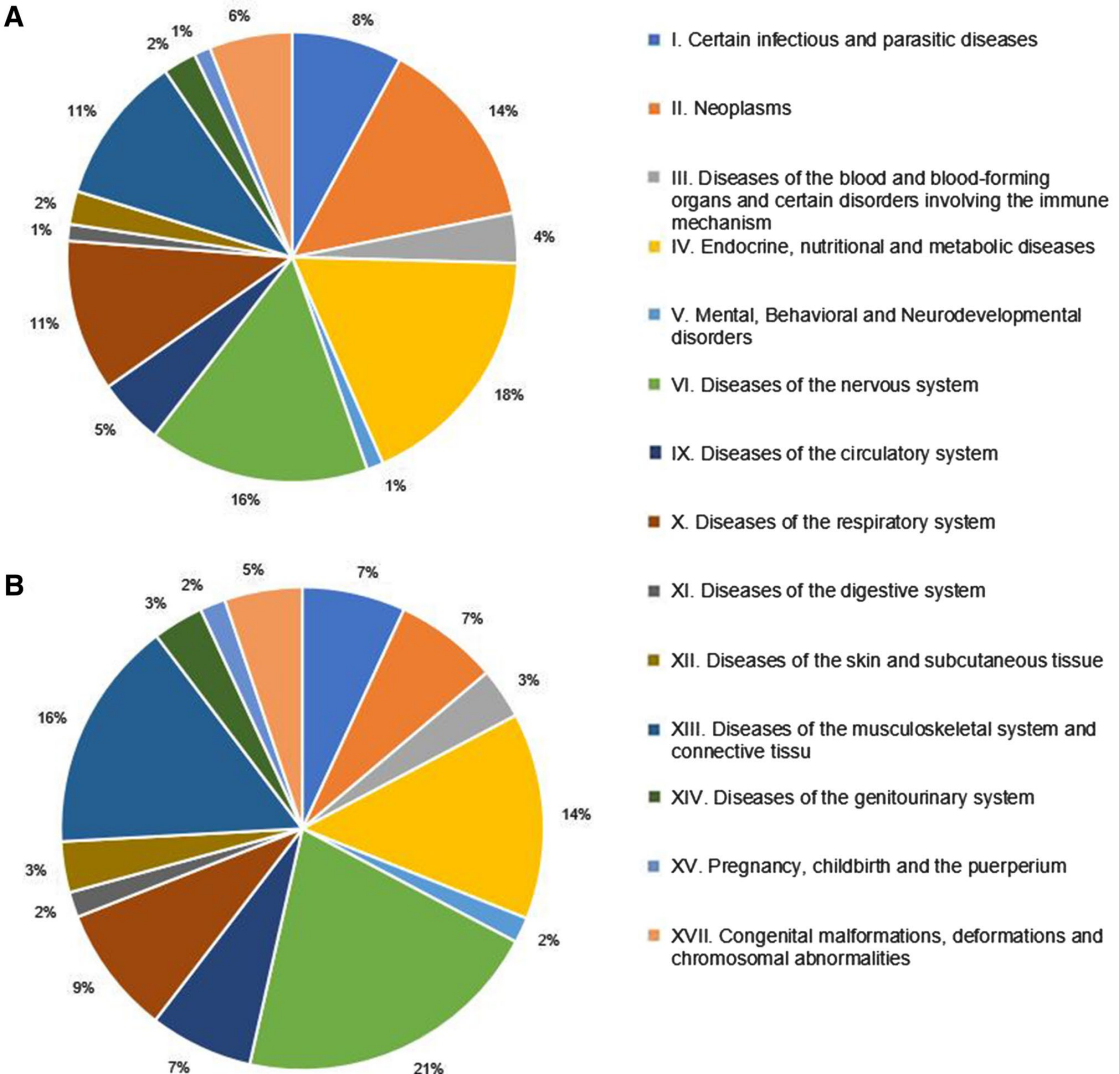
Distribution of COI studies by ICD classification. **a** Distribution of country-specific results across clinical areas defined by ICD groups (*n* = 83). **b** Distribution of studies between clinical areas defined by ICD groups (*n* = 58)

The majority of publications reported costs from one country (74%), but 15 studies presented results from multiple countries, and hence, altogether, 83 country-specific results were provided by 58 studies (Table 1). Three-quarters of the studies were published in English (*n* = 44), and except for five papers [18–22], all non-English papers had an English abstract. Most of the publications (n = 45, 78%) presented costs in euro. In 37 studies, the national currency was converted to euro; of them, 17 (46%) studies stated explicitly exchange rate, 5 (14%) studies reported only the source of exchange rate, and 15 (40%) studies did not mention conversion at all. Among countries outside the euro zone, reporting costs in national currency was most common in Romania (67%). Overall, 47 (81%) studies stated the source of funding. The lack of a funding statement was most prevalent in Romania (*n* = 2, 67%) and in Bulgaria (*n* = 5; 31%). Only two studies received funds from two different sources, both of them were funded by the European Union (EU) and the local government. Regarding clinical areas, endocrine, nutritional, and metabolic diseases were the most common, in which costs were analysed (*n* = 15 country-specific results), followed by neoplasms (*n* = 12), and certain infectious and parasitic diseases (*n* = 10) (Fig. 1). Altogether 48 different diseases were analysed in the 58 included articles.

**Table 1:** Characteristics of cost-of-illness studies

Characteristic	Number of country-specific results:* N* = 83; Number of papers:* N * = 58^1^									Total^a^
	Austria [24, 33, 58–64]	Bulgaria [18–22, 27–29, 32, 59, 65–70]	Croatia [27, 59, 71]	Czech Republic [23, 27, 34, 36, 59, 72–76]	Hungary [26–30, 35, 41, 59, 65, 67, 68, 75–86]	Poland [25, 27, 59, 75, 76, 87–92]	Romania [37, 59, 93]	Slovakia [59, 75, 76]	Slovenia [27, 31, 59, 94]	
Total number of studies	9	16	3	10	24	11	3	3	4	58
English	5	11	3	10	21	11	1	3	4	44
National language	4	5	0	0	3	0	2	0	0	14
Search										
Electronic database search	7	11	3	10	23	11	3	3	3	50
Hand-search	2	5	NA	NA	1	NA	NA	NA	NA	8
Currency										
Euro	9	10	3	10	21	10	1	3	3	45
National currency	NA	6	0	0	3	1	2	0	1	13
Source of resource use data										
Retrospective cross-sectional, self-completed questionnaire	6	9	0	3	15	1	0	0	0	28
Retrospective chart review	1	1	0	2	2	1	0	1	0	5
Interview-based prospective cohort	1	2	0	1	0	3	1	0	0	8
Retrospective claims data	0	0	0	1	5	3	1	1	0	8
Combination of various sources^b^	1	2	1	2	1	2	1	1	3	6
Modelling	0	1	2	1	1	1	0	0	1	2
NR	0	1	0	0	0	0	0	0	0	1
Perspective										
Public payer	2	2	2	2	2	3	0	1	2	10
Societal	2	8	0	3	18	4	0	0	1	30
Patient	2	0	0	0	0	0	0	0	0	2
Hospital	0	5	0	0	0	0	1	0	0	6
NR	5	1	1	5	4	4	2	2	1	13
Costing method										
Top–down	1	1	0	1	1	2	0	1	0	12
Bottom–up	3	10	1	3	16	2	0	1	2	22
NR	5	5	2	6	7	7	3	1	2	24
Indirect cost calculation method										
Human capital	5	8	0	3	18	7	0	1	1	34
Friction cost	1	1	1	2	1	1	1	1	1	11
NR	0	0	0	0	2	0	0	0	0	2
N/A	3	7	2	5	2	3	2	1	2	11
Informal care monetary valuation										
Proxy good	0	8	1	0	16	1	0	0	0	5
Opportunity cost	1	1	0	3	2	1	1	1	1	3
NR	2	0	0	2	0	1	0	0	0	20
Other	1	0	0	0	0	0	0	0	0	1
N/A	5	7	2	5	6	7	2	2	3	29
Funding source										
EU	1	8	0	0	9	1	0	0	0	13
Pharmaceutical industry	5	2	1	3	8	4	1	3	1	11
Government	1	0	0	5	3	1	0	0	0	13
Other	0	0	0	1	0	0	0	0	0	1
None	2	1	2	1	4	3	0	0	2	11
NR	1	5	0	0	0	3	2	0	1	11
Cost per patient reported										
Direct medical costs	5	13	1	4	20	5	3	1	1	38
Indirect costs	6	10	2	6	21	9	1	2	3	38
Informal care cost	4	9	1	5	18	3	1	1	1	29
Total costs	8	13	3	7	23	9	3	3	3	47
Any unit costs										
Reported	3	8	1	7	16	5	2	1	2	24
NR	6	8	2	3	8	6	1	2	2	34

*NR* not reported, *N/A* not applicable

^a^Several studies published results for multiple countries. These studies are referred in each relevant country columns in a row, while, in the total column, a study might be referred only once in a row. Therefore, adding numbers in a row results in a larger sum than in the total column

^b^Studies used combination of various sources of data: peer-reviewed published studies, national reports from governmental or professional bodies, extrapolations from similar countries, aggregated macrolevel data, claim data, and questionnaire survey

## Methods

Analyses by countries are presented in Table 1. The most frequently used data source was a retrospective, self-completed resource use questionnaire (48%), followed by retrospective claims data analysis (14%) and prospective diary (14%). Sample sizes ranged from *n* = 2 (small cohorts) to *n* = 127,512 (large population-based study). Of the 58 studies included in the review, 26 (45%) presented aggregated results for each main cost category (i.e., direct medical, direct non-medical, and indirect). The majority of studies applied the societal perspective (52%), followed by the public payer perspective (17%). If reported, bottom–up (38%) and top–down (21%) methods were used for estimating the costs in the studies. Productivity losses were estimated in 47 (81%) studies; of them, the human capital approach and friction cost method were used in 34 (72%) and 11 (23%) studies, respectively, and the method was not specified in 11 (23%) studies. Studies that reported costs of informal care (*n* = 29) applied the proxy-good method (17%) or the opportunity cost method (10%), but the name of the applied method was not stated in most of them (69%). Unit costs were not reported at all in 58% of the studies.

### Cost-of-illness: comparison across countries in one disease

Eighty-three COI estimates were reported for 48 different diseases. Apart from rare diseases, multiple sclerosis caused the highest economic burden in terms of average total annual cost per patient in three countries (Austria €50,599, the Czech Republic €14,777, and Poland €12,343) [23–25]. In Hungary, schizophrenia (€15,187), and in Bulgaria, gestational diabetes (€32,263) were the most costly diseases [22, 26].

Multi-country studies were conducted in nine diagnoses (rotavirus gastroenteritis, pneumonia, bladder cancer, hypoglycaemia, Duchenne muscular dystrophy, epidermolysis bullosa, Prader–Willi syndrome, cystic fibrosis, and haemophilia). One multi-country study (bladder cancer) was conducted in nine countries and another (hypoglycaemia) in six countries. Two studies were conducted (rotavirus gastroenteritis and pneumonia) in four countries and four studies (Duchenne muscular dystrophy, epidermolysis bullosa, Prader–Willi, and haemophilia) in two countries. The bladder cancer study involving nine countries resulted in mean total costs of €7421; however, costs differed significantly among countries, as the total cost was between €2320 (Bulgaria) and €16,479 (Slovenia). The direct medical cost ranged between €1090 (Bulgaria) and €8050 (Slovenia), and indirect cost varied between €912 (Bulgaria) and €6398 (Slovenia). The hypoglycaemia study was conducted in six countries, and the total overall societal cost per patient with diabetes was €11 and ranged between €5 (Bulgaria) and €18 (Slovenia) [27]. Rotavirus gastroenteritis and pneumonia studies were conducted in four countries and the average total costs were €541 and €764, respectively. Costs varied between €494 (Czech Republic) and €747 (Poland) in rotavirus gastroenteritis, and between €472 and €1111 in pneumonia. Duchenne muscular dystrophy, epidermolysis bullosa, Prader–Willi syndrome, cystic fibrosis, and haemophilia were studied in two countries (Hungary and Bulgaria) applying the same methodology in a European Commission founded rare disease study (BURQOL-RD project). Prader–Willi syndrome was the least costly (Bulgaria: €3842 Hungary: €12,532) and mucopolysaccharidosis was the most costly rare disease (Bulgaria: €77,414; Hungary: €25,326) [28, 29].

Unique studies in more than one country were conducted in eight diagnoses, namely multiple sclerosis, dementia, Parkinson’s disease, rheumatoid arthritis, osteoporosis, chronic obstructive pulmonary disease (COPD), systemic sclerosis, and diabetes. Multiple sclerosis and diabetes were studied most often (four studies each), while three unique studies in three different countries were conducted in Parkinson’s disease and two unique studies in three different countries were conducted in cystic fibrosis. Two unique studies on both dementia and COPD were conducted in two different countries. In multiple sclerosis, there was a 4.1 times difference in total costs between Austria (€50,599) and Poland (€12,343) [24, 30]. In diabetes, the highest direct cost was observed in Hungary (€1309) and the lowest total cost was observed in Bulgaria (€472) [31, 32]. In Parkinson’s disease, there was a 3.3 times difference in total costs between Austria (€22,984) and the Czech Republic (€6970) [33, 34]. In dementia, we found a 3.5 times difference in total costs between the Czech Republic (€2013) and Hungary (€671) [35, 36]. The costs of COPD were similar in Bulgaria (€1839) and Romania (€2103) [21, 37].

**Fig. 2:**
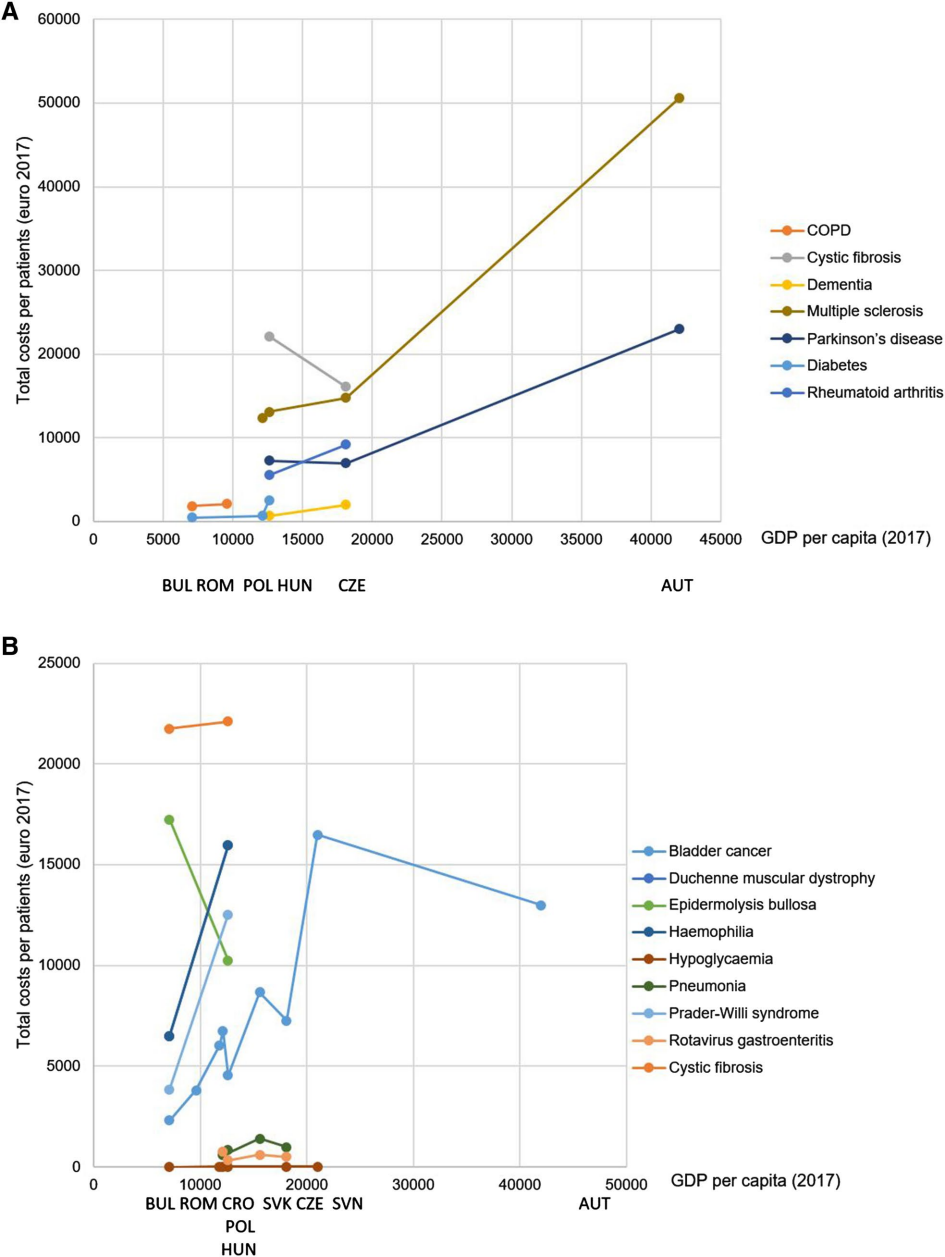
Total costs (euro 2017) and GDP per capita (2017): comparison of single-country and multi-country studies. **a** Single-country studies: each line represents one disease, and each dot represents one study and one country. **b** Multi-country studies: each line represents one study and one disease, and each dot represents one country

Adjusting costs for GDP per capita level, differences between countries decreased (Table 2). For instance, a 7.1-fold difference in bladder cancer and a 4.1-fold difference in multiple sclerosis were reduced to 2.4- and 1.5-fold, respectively. Comparing diseases with available cost estimates from more than one country (Fig. 2), a positive relationship was identified between costs and GDP per capita.

**Table 2:** Cost-of-illness in nine CEE countries (€ 2017)

Disease	Country	Study	Costing year	Sample size	Perspective	Resource use data source	EUR/patient/year converted to € 2017				Total cost as % of GDP/capita
							Total costs	Direct medical	Direct non-medical	Indirect costs	
I. Certain infectious and parasitic diseases (ICD A00–B99)											
Acute gastrointestinal infections	POL	Czech et al. [87]	2009	NR	Societal	Interview-based prospective cohort, follow-up period = 4 weeks	196	77	16	103	1.7%
Clostridium difficile infection	HUN	Kopcsóné Németh et al. [95]^a^	2011	151	Hospital	Retrospective chart review	656–1397	NR	NR	NR	5.2–11.1%
HIV infection	AUT	Grabmeier-Pfistershammer et al. [58]	2006	24	NR	Retrospective chart review	28,572	NR	NR	NR	5.7%
Rotavirus gastroenteritis	CZE HUN POL SVK	Tichopad et al. [75]	2013	109 NR 112 115	Payer	Retrospective chart review	494 324 747 597	NR NR NR NR	NR NR NR NR	NR NR NR NR	2.7% 2.6% 6.2% 3.8%
II. Neoplasms (C00–D48)											
Bladder cancer	AUT BUL HRV CZE HUN POL ROU SVK SVN	Leal et al. [59]	2012	NR	NR	Publicly available sources and claims data were combined	12,988 2320 6035 7266 4545 6757 3812 8677 16,479	7965 1090 2520 4511 2748 3466 1750 6143 8050	NR	3292 912 2725 1935 1061 2333 1548 1749 6398	30.9% 32.7% 51.1% 40.1% 36.1% 55.8% 39.7% 55.6% 78.5%
Breast cancer	HUN	Inotai et al. [41]	2012	127,512	NR	Retrospective claims data	1622	NR	NR	NR	12.9%
Cervical cancer	POL	Dubas-Jakóbczyk et al. [88]	2012	NR	Societal	Publicly available sources and social insurance data were combined	NR	NR	NR	8457,898^f^	NA
Colorectal cancer	HUN	Inotai et al. [41]	2012	118,235	NR	Retrospective claims data	2010	NR	NR	NR	16.0%
Lung cancer	HUN	Inotai et al. [41]	2012	126,731	NR	Retrospective claims data	2663	NR	NR	NR	21.1%
Prostate cancer	HUN	Inotai et al. [41] Brodszky et al. [40]	2012 2005	56,382 17,642	Payer Payer	Retrospective claims data Retrospective follow-up cohort of claims data, follow-up = 8 years	1656 12,072	NR NR	NR NR	NR NR	13.1% 95.8%
Skin melanoma	HRV	Bencina et al. [71]	2011	NR	Payer	Modelling	Stage 0: 104–stage 4: 4610	NR	NR	NR	1.0-39.1%
VI. Diseases of the nervous system (G00–G99)											
Alzheimer’s disease	CZE	Maresova et al. [73]	2014	NR	NR	Publicly available sources and claims data were combined	NR	13,208		NR	73.0%
Dementia	HUN CZE	Érsek et al. [35]. Holmerová et al. [36]	2008 2014	88 119	Societal NR	Cross-sectional self-completed questionnaire Cross-sectional self-completed questionnaire	671 2013^b^	222	387	63 1769^b^	5.3% 11.1%
								238^b^			
Epilepsy	HUN	Péntek et al. [83]	2009	100	Societal	Cross-sectional self-completed questionnaire	2650	885	465	1300	21%
Multiple sclerosis	AUT CZE HUN POL	Kobelt et al. [24] Dusankova et al. [23] Péntek et al. [30] Szmurlo et al. [25]	2005 2007 2009 2012	1019 909 68 NR	Societal Societal Societal Societal	Cross-sectional self-completed questionnaire Prospective cohort, follow-up = 3 ms Cross-sectional self-completed questionnaire Extrapolation from other country	50,599 14,777 13,115 12,343	21,788 7581 8744 5805	10,109 550 1576 510	18,399 6646 2696 6028	120.5% 81.6% 104.1% 102.0%
Parkinson’s disease	AUT CZE HUN	Campenhausen et al. [33] Winter et al. [34] Tamás et al. [85]	2008 2004 2009	81 100 110	Societal Societal Societal	Cross-sectional self-completed questionnaire Cross-sectional self-completed questionnaire Cross-sectional self-completed questionnaire	22,984 6970 7257	13,833 4238		9151 2733 2534	30.9% 38.5% 57.6%
								2586	2136		
IX. Diseases of the circulatory system (I00–I99)											
Acute myocardial infarction	HUN	Gulácsi et al. [80].	2003	996	Societal	Claims data	NR	NR	NR	947	7.5%
Chronic heart failure	POL	Czech et al. [92]	2010	400	Public payer	Interview-based prospective cohort, follow-up period = 4 weeks	1991	NR	NR	NR	16.5%
Coronary artery disease	POL	Jaworski et al. [89]	2005	2593	NR	Cross-sectional self-completed questionnaire	2851	1365	NR	1486	23.6%
Subarachnoide bleeding	BUL	Georgieva et al. [18]^a^	2014	61	Hospital	Prospective cohort	NR	3685	NR	NR	51.9%
X. Diseases of the respiratory system (J00–J99)											
Bronchial Asthma	BUL	Ivanova et al. [20]^a^	2014	112	Hospital cost	Retrospective chart review	200–393^c^	200–393^c^	NR	NR	2.8-5.5%
COPD	BUL ROU	Kyuchukov et al. [21]^a^ Stâmbu et al. [37]	NR 2006	84 85	Hospital and patient NR	Prospective cohort Interview data	1839 2103	898 2103	NR NR	NR NR	25.9% 21.9%
Lower respiratory tract infection	BUL	Glogovska et al. [19]^a^	NR	1441 ambulatory + 353 hospitalized	Health system	NR	NR	1218	NR	NR	17.2%
Pneumonia	CZE HUN POL SVK	Tichopad et al. [76]	2010	258 NR 198 315	NR	Claims data	Ages 50–64/> 65 1194/786 1009/686 714/472 1685/1111	Ages 50–64/age > 65 708/786 686/686 472/472 1190/1111		Ages:50-64/> 65 486/0 323/0 242/0 495/0	6.6%/4.3% 8.0%/5.4% 5.9%/3.9% 10.8%/7.1%
*Streptococcus pneumoniae*	ROU	Stoicescu et al. [93]	2004	48,200	Public payer	Claims data	8.3 million	8.3 million	NR	NR	NA
XIII. Diseases of the musculoskeletal system and connective tissue (M00.0–M99.9)											
Chronic non-specific back pain	AUT	Wagner et al. [64]^a^	2008	48	Public payer	Retrospective self-completed questionnaire	2148	1687	461	NR	5.1%
Osteoporosis	SVN AUT	Dzajkovska et al. [94] Dimai et al. [62]	2003 2008	NR 441/population-based	Societal NR	Publicly available sources and claims data were combined Publicly available sources and retrospective self-completed questionnaire were combined	34,524,727^d^ 827,849,562^d^	24,432,069^d^ 520,419,423^d^ 1	10,092,657^d^ 307,430,139^d^	NA NA	
Osteoarthritis of hip and knee	AUT	Wagner et al. [63]	2008	174	Public payer	Retrospective self-completed questionnaire	3211	1342	1869	NR	7.6%
Rheumatoid arthritis	CZE HUN	Klimes et al. [72] Péntek et al. [86]	2014 2004	261 255	Societal NR	Cross-sectional self-completed questionnaire Cross-sectional self-completed questionnaire	9176 5536	7442		1733 3034	50.7% 43.9%
								1524	978		
Systemic lupus erythematosus	POL	Kawalec et al. [90]	2012	1600	NR	Claims data	NR	NR	NR	1363	11.2%
Systemic sclerosis	POL HUN HUN	Kawalec et al. [90] Lopez Basida et al. [28] Minier et al. [82]	2012 2012 2006	500 38 80	NR Societal Societal	Claims data Cross-sectional self-completed questionnaire Cross-sectional self-completed questionnaire	NR 4822 13,769	NR 1272 4724	NR 1184 1330	3394 2366 7716	28.0% 38.3% 109.3%
IV. Endocrine, nutritional and metabolic diseases (E00–E90)											
Diabetes	BUL POL HUN SVN	Valov et al. [32] Lesniowska et al. [91] Brodszky et al. [78] Nerat et al. [31]	2011 2009 2003 2011	433 NR 480 NR	Payer Societal NR Payer	Retrospective and prospective cohort, follow-up = 6 ms Claims data Cross-sectional self-completed questionnaire Publicly available sources	472 659 2514 NR	NR 287 1309 882		NR 152 1118 NR	6.6% 5.4% 20.0% 4.2%
Hypoglycaemia	HUN BUL HRV CZE POL SVN	Jakubczyk et al. [27]	2013 2014 2012 2011 NR 2011	NR	Public payer/societal	Modelling	9.8 5.4 7.5 10.9 11.3 17.7	7.2 4.7 6.7 9.2 9.5 15.2		2.6 0.7 0.8 1.7 1.8 2.5	0.1% 0.1% 0.1% 0.1% 0.1% 0.1%
Other top level ICD items including < 2 disease											
Benign prostatic hyperplasia	HUN	Rencz et al. [84]	2014	246	Societal	Cross-sectional self-completed questionnaire	902	417	275	210	7.2%
Endometriosis	AUT	Prast et al. [60]	2009	73	Healthcare system	Cross-sectional self-completed questionnaire	8945	6501		2443	21.3%
Gastro-oesophageal reflux disease	AUT	Willich et al. [61]	2000	5273	NR	Prospective cohort, follow-up = 4 yrs	527	471		55	1.3%
Gestational diabetes	BUL	Todorova et al. [22]	2002-2005	195	Healthcare system	Cross-sectional self-completed questionnaire	32,263	32,263	NR	NR	454%
Psoriasis	HUN	Balogh et al. [77]	2013	200	Societal	Cross-sectional self-completed questionnaire	9524	7816	152	1292	75.6%
Psoriatic arthritis	HUN	Brodszky et al. [78]	2007	183	Societal	Cross-sectional self-completed questionnaire	7395	2489	1053	3853	58.7%
Sarcoidosis	POL	Kawalec et al. [90]	2012	2700	NR	Claims data	NR	NR	NR	1114	9.2%
Schizophrenia	HUN	Péntek et al. [26]	2009	78	Societal	Cross-sectional self-completed questionnaire	15,187	4334	819	10,034	120.5%
Rare diseases											
Cystic fibrosis	BUL CZE HUN BUL	Iskrov et al. [70] Mlcoch et al. [74] Chevreul et al. [68] Chevreul et al. [68]	2012 2010 2012 2012	33 330 110 33	Societal NR Societal Societal	Cross-sectional self-completed questionnaire Retrospective registry analysis Cross-sectional self-completed questionnaire Cross-sectional self-completed questionnaire	23,570^b^ 16,118 22,121 21,759	18,551^b^ 16,118 20,393 21,176		0^b^ NR 3802 1068	332.0% 89.0% 175.6% 306.5%
Duchenne muscular dystrophy	HUN BUL	Cavazza et al. [67]	2012	57 14	Societal	Cross-sectional self-completed questionnaire	15,952 6500	15,094 2289	712 4211	145 0	126.6% 91.5%
Epidermolysis bullosa	BUL HUN	Angelis et al. [96]	2012	8 6	Societal	Cross-sectional self-completed questionnaire	17,246 10,262	3503 438	13,485 9823	259 0	242.9% 81.4%
Fragile X syndrome	HUN	Chevreul et al. [79]	2012	12	Societal	Cross-sectional self-completed questionnaire	5180	116	5065	0	51.6%
Haemophilia	BUL HUN	Cavazza et al. [66]	2012	20 58	Societal	Cross-sectional self-completed questionnaire	6500 15,952	2289 15,094	2326 158	0 145	91.5% 126.6%
Histiocytosis	BUL	Iskrov et al. [69]	2012	7	Societal	Cross-sectional self-completed questionnaire	6668	1657	2865	2145	93.9%
Mucopolysaccharidosis	BUL HUN	Péntek et al. [29]	2012	2 10	Societal	Cross-sectional self-completed questionnaire	77,414 25,326	46,229 699	31,185 19,862	0 5091	1090.3% 201.0%
Prader–Willi syndrome	BUL HUN	Lopez Basida et al. [28]	2012	8 5	Societal	Cross-sectional self-completed questionnaire	3842 12,532	2489 325	1354 12,207	0 0	54.1% 99.5%

^a^Study identified through hand-search of local, non-indexed journals

^b^Median

^c^Bronchial asthma + exacerbations, bronchial asthma + pneumonia, and bronchial asthma + bronhiectasia

^d^Aggregated costs for the total population of patients

## Discussion

A systematic search was conducted to provide a review of the COI studies in nine CEE countries. The diffusion of the new technologies to the health scare systems is enormous, prices, and technologies, and professional guidelines are changing; therefore, our search was limited for the past 10 years. The included papers covered a broad range of clinical areas and showed notable cross-country differences in terms of methodology and publication standards as well as the average yearly costs per patient.

### Study characteristics and methodology

Reporting cost results in euros was dominant over national currencies, suggesting that researchers in the CEE region find it important to make their results available for the international scientific community and allow for comparability with other studies. To assess study quality, we selected some quality indicators, such as those are used in health economics checklists. Reporting study perspective, reference year, costing method (top–down vs. bottom–up), source of resource use, valuation of informal care, valuation of productivity loss, and funding source were considered as quality indicators. We find it noteworthy to mention that whilst the source of data on resource utilization and reference year of costing were stated in nearly every paper (98% and 95%, respectively), other important quality indicators were less often reported. The study perspective was reported in 78%, the approach to valuing indirect costs in 77%, costing method in 64%, at least one unit cost in 42%, and method for valuing informal care in 31% of the studies. A recent review of economic evaluations in Austria found that the study perspective and reference year were not reported by 60% and 25% of the studies, respectively [13]. Differences may be explained by inclusion of non-peer-reviewed or grey literature (e.g., economic evaluation reports from national health technology assessment agencies) and of other forms of economic evaluations in the study by Mayer et al. The review by Mayer et al. included 93 (partial and full) economic evaluations, 14 of which were cost-of-illness analyses. Out of the 93 included studies, 23 were not indexed according to the Journal Citation Reports (Social) Sciences Edition and 12 were non-peer-reviewed reports [13].

### Clinical areas

A large variety of diseases was covered by the studies, and most of them occurred in a one study. Each disease was studied by, on average, 1.3 papers. Considering country-specific results by ICD categories, endocrine, nutritional, and metabolic diseases (18%), neoplasms (14%), infectious (12%), neurologic (11%), and musculoskeletal diseases (11%) represented the five main fields of COI research in CEE. It is difficult to judge the drivers of the selection of clinical fields. The public health importance of a disease might be an important factor as, for instance, all the studies in the ‘Endocrine, nutritional and metabolic diseases’ ICD category were related to diabetes, and among neoplasms studies, the most prevalent malignancies (breast, colorectal, lung, and prostate cancer) were present (Table 2). According to the Global Burden of Disease study, the leading three causes of total Disability-Adjusted Life Years (DALY) included ischaemic heart disease, cerebrovascular disease, and lower respiratory infection, comprising 16% of all DALYs [38]. Leading causes of DALYs were represented only in six (10%) studies (cerebrovascular disease: *n* = 1, ischaemic heart disease: *n* = 2, and lower respiratory infection: *n* = 3) in our review, questioning public health importance as a driver of topic selection in COI studies. The need for COI data to support decision-making on reimbursement of highly effective but costly new drugs seems to be another relevant issue, and this hypothesis is supported by the relatively high rate of studies in inflammatory rheumatic diseases, where biological drugs were introduced in the CEE countries in the observed period. Multiple sclerosis is another disorder where biologicals revolutionized the treatment that partly explains the relatively high rate of neurological studies in the region. Moreover, when counting papers, neurologic diseases were most frequently studied (19%). A possible explanation could be that neurologic conditions in the CEE region were priorities for state-funded or EU-funded research. Eight out of the ten COI studies focusing on neurologic diseases received funding from the local governments or EU organisations. It is interesting that neurologic diseases were found also the most frequently studied clinical area according to a recently published systematic review of EQ-5D studies in the CEE region [39]. These results suggest that neurologic diseases have a high priority in health economics research in the CEE.

### Comparison of costs across countries

With respect to diseases for which cost estimates were present in multiple countries, costs varied substantially across countries. However, there are apparent differences in the level of comparability between studies. There were multi-country studies following a standardized methodology in which more than one CEE country together with Western European countries was participated. We also identified single-country studies in various diseases using very different methods. Both multi-country and single-country studies reported significant cost differences in diseases across countries.

For the interpretation of data, it is important to take into consideration that the number of patients, sample characteristics (e.g., age, gender, disease duration, and disease severity), and the availability of costly treatments at the time of the study (e.g., biological drugs for inflammatory diseases) varied a great deal across studies that may strongly influence the COI results and their comparability. Large differences in unit costs can also cause significant variations in costs. In bladder cancer, for example, the cost of an inpatient day was seven times higher in Austria (€495) than in Romania (€67). Methodological differences, such as prevalence- and incidence-based costing, form an obstacle for the comparison of costs. Therefore, the incidence-based prostate cancer study by Brodszky et al. cannot be compared with the prevalence-based prostate cancer study by Inotai et al., although both studies were conducted in Hungary [40, 41]. It should also be noted that differences in health care systems (private/public, financing, etc.) might have a significant impact on costs; for instance, global budget, fee-for-service or DRG financing mechanisms, the presence of co-payments, minor or major share of private services, and many more aspects might influence the actual costs, access to health care, and, finally, the COI figure [42].

According to the literature, one might expect a higher COI in a country with a higher GDP [43–45]. In many diseases (multiple sclerosis, bladder cancer, Parkinson’s disease, rheumatoid arthritis, Prader–Willi syndrome, haemophilia, diabetes, and hypoglycaemia), there was a clear positive association between total costs and GDP per capita. As opposed to this, cost estimates, sometimes, inversely correlated with the per capita GDP. For instance, GDP per capita in Bulgaria is almost half of that in Hungary; nevertheless, costs of mucopolysaccharidosis were threefold higher in Bulgaria. Thus, in some cases, adjusting costs for the GDP further increased the inter-country differences. On the other hand, the 3.5-fold higher GDP per capita in Austria decreased the cross-country differences (from 4- to 1.3-fold) in costs of multiple sclerosis. In spite of the considerable heterogeneity observed in the studies included in this review, some trends could be identified. The magnitude of costs increased with the level of per capita GDP. In other words, cross-country differences decreased or even vanished when the costs were adjusted. In contrast, higher costs with lower GDP per capita could be observed only in some rare diseases (cystic fibrosis, epidermolysis bullosa, and mucopolysaccharidosis) and rotavirus gastroenteritis. Moreover, methodological differences did not seem to affect this relationship. Comparing multi-country studies in a disease applied the same methodology for more than one country and single-country studies analysed costs in the same disease, the relationship between cost-of-illness and GDP per capita showed similar pattern in these two groups of studies (see Fig. 2).

### Quality, publication standards, and the assessment of transferability

Cost-of-illness studies varied considerably both in methods and in cost estimates, and serve many purposes. Methodological deficiencies, such as the lack of reporting either on the three distinct phases of costing (identifying the relevant cost items, measuring the use of the identified resources, and placing a value on these cost items) [46], or other important characteristics such as the perspective of the study, related to the production function (direct and indirect costs) were the leading causes of shortcomings in comparability. However, no specific costing guidelines for health care interventions are available in these countries, and except in Austria, there is no national cost database available, providing some kind of unit cost data in a collected form [13, 47, 48]. Another important difficulty in costing relates to the different Managed Entry Agreements (MEA), such as price volume agreements, discounts, outcome guarantees, and many more, in the reimbursement of the health technologies in the different countries [49, 50]. Due to the MEAs, for instance, the real purchasing price of the medicinal products is not publicly available.

Several papers were published about transferability in the past 2 decades [51–56]. At the moment, health economics and health technology assessment guidelines in CEE countries either include very limited advice or provide no guidance on the transferability or adaptation of clinical and economic data from other jurisdictions. Thus, establishing better guidelines for COI studies on transferability would be valuable for robust decision-making in the CEE countries [56]. As Gao et al. stated, confirming the transferability of COI estimates across jurisdictions would contribute significantly to resolving the issue of transferability of cost-effectiveness results [45]. Transferability is a very important issue around the world and especially in Central or Eastern Europe with limited resources to provide COI studies [53–55]. Data transferability and transferability of the results are not discussed in these COI studies. Both should be improved using Drummond’s check list for evaluating economic evaluations [57]. Transferability might be an important alternative to conduct local COIs. However, due to the methodological, data, and publication heterogeneity, the usefulness of the COI results in other jurisdictions is limited.

### Limitations

There are a few limitations to note. A systematic approach was taken to identify studies that have considered the costs of diseases; however, the possibility that relevant studies were not identified and included in this systematic literature review remains. Some COI results might have been missed due to excluding grey literature (i.e., conference abstracts and project reports) from our search. Other limitationis that the local search in non-indexed journals was conducted only in three of the nine countries. On the other hand, no language restriction was applied in the systematic search. Adopting a Medical Subject Heading (MeSH)-based search strategy may have led to missing some studies using keywords improperly. At the same time, the PubMed search engine uses a broad range of entry terms which may minimize the number of excluded studies. Further limitation is that no comprehensive checklist was applied, because, according to our best knowledge, there is no COI study-specific checklist in English. This might bias our conclusions on study quality, but we believe that the presented study characteristics could give a good overall description of the included studies.

## Conclusions

Fifty-eight COI studies were identified between 1 January 2006 and 30 June 2017 published in Austria, Bulgaria, the Czech Republic, Croatia, Hungary, Poland, Romania, Slovakia, and Slovenia, providing 83 country-specific COI results. Endocrine, nutritional, and metabolic diseases, neoplasms, infectious disease, and neurological disorders were the most frequently studied clinical areas. Transferability might be an important alternative to conduct local COIs. However, due to the methodological, data, and publication heterogeneity of these 58 COI studies, the transferability is limited across the nine Central and Eastern European Countries.

## Electronic supplementary material

Below is the link to the electronic supplementary material.

Supplementary material 1 (DOCX 60 kb)
