# Supplementary material for: Cost-of-illness studies in nine Central and Eastern European countries
Source: Eur J Health Econ. 2019 May 18;20(Suppl 1):155–72. doi: 10.1007/s10198-019-01066-x (PMC6544593; doi:10.1007/s10198-019-01066-x)
Supplement: Supplementary file 1 — Supplementary material 1 (DOCX 60 kb) [file 10198_2019_1066_MOESM1_ESM.docx]

**Appendix**

**Cost-of-illness studies in nine Central and Eastern European countries**

Valentin Brodszky, Zsuzsanna Beretzky, Petra Baji, Fanni Rencz, Márta Péntek, Alexandru Rotar, Konstantin Tachkov, Susanne Mayer, Judit Simon, Maciej Niewada, Rok Hren, László Gulácsi

**Search term for MEDLINE via Pubmed**

"cost of illness"[MeSH Terms] AND ("Hungary"[All Fields] OR "Bulgaria"[All Fields] OR "Croatia"[All Fields] OR "Slovenia"[All Fields] OR "Slovakia"[All Fields] OR "Romania"[All Fields] OR "Poland"[All Fields] OR "Austria"[All Fields] OR "Czech Republic"[All Fields]) AND ("2006/01/01"[PDAT] : "2017/06/01"[PDAT])

**Figure S1 Study selection, PRISMA flowchart**

Records identified through database searching
(n =853)

Medline (n=312)

The Cochrane Library (n=77)

CINAHL (n=8)

Web of Science (n=141)

EMBASE (n=315)

Records excluded by publication type (n=153)

Abstract (n=55)

Review (n=98)

Studies included
(n =58)

Abstract or full-text articles assessed for eligibility
(n =461)

)

Records screened
(n =614)

Records after duplicates removed
(n = 614)

## Included

## Eligibility

## Screening

## Identification

Articles excluded by
the eligibility criteria (n=403)

Doesn’t include cost of disease data (n=282)

Not one specific disease

(n=54)

Not cost of disease (treatment related cost, cost-effectiveness analysis, budget impact analysis

(n=67)

Additional records identified through hand search
(n=8)

**Table S3**

**Costs-of-illness studies applying restriction on disease population or treatments in nine CEE countries (€)**

| **Disease** | **Country** | **Study** | **Costing year** | **Sample size** | **Perspective** | **Resource use data** | **Cost** |
| --- | --- | --- | --- | --- | --- | --- | --- |
| Crohn’s disease surgical cost  (mean hospital cost) | POL | Keller et al. 2013 [1] | NR | 92 | public payer | retrospective chart review, hospital database | 6,882 laparoscopic, 8,180 open, and 10,085 for converted cases |
| Disease-related undernutrition in chronic diseases^a^  (average direct cost per patient) | HRV | Benkovic et al. 2014 [2] | 2011 | 114,220 | public payer | claims data | 1,745 |
| Posttransplant events in renal transplant recipients  average 3-year total cost | CZE  POL | Chamberlain et al. 2014 [3] | NR | 2,818 | public payer | claims data | 34,720  39,561 |
| Rheumatoid arthritis, long-term care, cost/patient/year | HUN | Horváth et al. 2014 [4] | 2012 | 976 | public payer | claims data | 744 |
| Indirect cost (absenteeism) of six chronic diseases^b^  cost/patient | POL | Malinowski et al. 2016 [5] | 2012 | 45,000 | societal | social insurance claims data | 650 (psoriasis) - 5,831 (multiple sclerosis) |
| Indirect cost (absenteeism) of three chronic diseases^c^  cost/patient | POL | Kawalec et al. 2015 [6] | 2012 | 4,800 | societal | social insurance claims data | 1,117 (sarcoidosis) – 3,395 (systemic lupus erythematosus) |
| COPD in out patient care  total cost per patient per year^d^ | POL | Jahnz-Różyk et al. 2011 [7] | 2007-2008 | 107 | societal | claims data | 1,187 |
| Stroke, direct hospital cost in the first and second 12 months of active and chronic care | HUN | Kárpáti 2007 et al. [8] | 2003-2005 | 3,535 new stroke patients | public payer | claims data | first 12 months: 1,736 (acute care) and 239 (chronic)  next 12 months: 562 (acute) and 134 (chronic) per patient |
| Influenza annual cost in elderly population | CZE  HUN  POL  ROU | Kovács et al. 2014 [9] | 2009-2011 | NR | NR | claims data | 661,473  1,333,875  5,955,149  410,571  (total cost) |
| Multiple sclerosis direct and indirect costs of two treatment regimens A and B^e^ | POL | Matschay et al. 2008 [10] | 2004-2005 | 120 | societal | retrospective hospital database | 24,688 (group A) and 393 (group B) |
| Metastatic colorectal carcinoma; direct medical cost systemic therapy / patient/year | SVN | Mesti et al. 2015 [11] | 2009 | 294 | public payer | retrospective hospital data | 22,656 |
| Direct annual average costs of hypertension treatment  among adolescent | POL | Paczkowska et al. 2014 [12] | 2010 | 480 | societal | claims data | 98.47 |
| Radiation therapy of localized prostate cancer  cost /intervention /patient in A, B and C therapy^f^ | HUN | Zemplényi et al. 2016 [13] |  | NR | healthcare provider | retrospective hospital data | 2,038 (A)  3,072 (B)  2,278 (C) |

a Undernutrition associated with tumour cachexia, chronic pancreatitis, inflammatory bowel disease, hepatic encephalopathy, chronic obstructive pulmonary disease, chronic renal insufficiency requiring dialysis, cerebrovascular insult, pressure ulcers, and femoral fractures in the elderly. Average direct costs/patient are between 544.87 EUR (femur fracture) and 3531.62 (osophageal carcinoma)

b Seropositive rheumatoid arthritis, other types of rheumatoid arthritis, psoriasis, multiple sclerosis, Type 1 diabetes, and ulcerative colitis. Total cost is available, indirect cost is calculated using Gross income per worker (in the Table) Gross Domestic product/capita and Gross Value Added per worker. The cost per patient varied from EUR 900 for psoriasis to 8000 for multiple sclerosis, calculated using GVA per worker

c Systemic lupus erythematosus, systemic sclerosis and sarcoidosis. Total cost is available, indirect cost is calculated using Gross income per worker (in the Table) Gross Domestic product/capita and Gross Value Added per worker.

d The total cost per patient per year was 4027.82 zlotys (1007 euro) and included the cost of chronic treatment in the amount of 2423.57 zlotys (606 euro) plus the cost of treatment of an acute exacerbation in the outpatient setting in the amount of 421.16 zlotys (105 euro) plus the cost of treatment of an acute exacerbation in the inpatient setting in the amount of 1183.09 zlotys (296 euro).

e Group A– patients receiving continuous interferon therapy (Betaferon) and steroids during relapses, and Group B – patients receiving steroid-only (Solu-Medrol, Metypred) treatment)

f Average treatment delivery times were 14.5 minutes for three-dimensional radiation therapy, 16.2 minutes for intensity-modulated radiation therapy with image-guided and 14 minutes without image-guided method. Expected mean cost of patients undergoing conventional three-dimensional radiation therapy, normal and hypofractionated intensity-modulated radiation therapy were 619 000 HUF, 933 000 HUF and 692 000 HUF, respectively. A: conventional three-dimensional radiation therapy; B: normal; C: hypofractionated intensity-modulated radiation therapy.

**Reference list**

1. Keller DS, Katz J, Stein SL, Delaney CP. Surgical cost of care in Crohn's disease. Pol Przegl Chir. 2013; 85(9): 511-6.

2. Benkovic V, Kolcic I, Ivicevic Uhernik A, et al. The economic burden of disease-related undernutrition in selected chronic diseases. Clin Nutr. 2014; 33(4): 689-93.

3. Chamberlain G, Baboolal K, Bennett H, et al. The Economic Burden of Posttransplant Events in Renal Transplant Recipients in Europe. Transplantation. 2014; 1.

4. Horvath CZ, Sebestyen A, Osterle A, et al. Economic burden of long-term care of rheumatoid arthritis patients in Hungary. Eur J Health Econ. 2014; 15 Suppl 1S131-5.

5. Malinowski KP, Kawalec PP, Mocko P. Indirect costs of absenteeism due to rheumatoid arthritis, psoriasis, multiple sclerosis, insulin-dependent diabetes mellitus, and ulcerative colitis in 2012: a study based on real-life data from the Social Insurance Institution in Poland. Expert Rev Pharmacoecon Outcomes Res. 2016; 16(2): 295-303.

6. Kawalec PP, Malinowski KP. The indirect costs of systemic autoimmune diseases, systemic lupus erythematosus, systemic sclerosis and sarcoidosis: a summary of 2012 real-life data from the Social Insurance Institution in Poland. Expert Rev Pharmacoecon Outcomes Res. 2015; 15(4): 667-73.

7. Jahnz-Rozyk K, Targowski T, From S, Faluta T, Borowiec L. [Costs of chronic obstructive pulmonary disease in patients treated in ambulatory care in Poland]. Pneumonol Alergol Pol. 2011; 79(5): 337-42.

8. Karpati K, Majer I, Boncz I, et al. [Social insurance costs of stroke hospital treatments in Hungary; 2003–2005]. Ideggyogy Sz 2007; 60(7-8): 311-20.

9. Kovacs G, Kalo Z, Jahnz-Rozyk K, et al. Medical and economic burden of influenza in the elderly population in central and eastern European countries. Hum Vaccin Immunother. 2014; 10(2): 428-40.

10. Matschay A, Nowakowska E, Hertmanowska H, Kus K, Czubak A. Cost analysis of therapy for patients with multiple sclerosis (MS) in Poland. Pharmacol Rep. 2008; 60(5): 632-44.

11. Mesti T, Boshkoska BM, Kos M, Tekavcic M, Ocvirk J. The cost of systemic therapy for metastatic colorectal carcinoma in Slovenia: discrepancy analysis between cost and reimbursement. Radiol Oncol. 2015; 49(2): 200-8.

12. Paczkowska A, Koligat D, Nowakowska E, Hoffmann K, Bryl W. Analysis of direct costs of hypertension treatment among adolescents in Poland. Acta Pol Pharm. 2014; 71(1): 197-203.

13. Zemplenyi AT, Mangel L, Kalo Z, Endrei D, Boncz I. [A microcost analysis of radiation therapy of localized prostate cancer]. Orv Hetil. 2016; 157(12): 461-8.
